# The membrane skeleton density of red blood cells in *MYH9*-related disease patients is decreased

**DOI:** 10.1042/BSR20260386

**Published:** 2026-09-29

**Authors:** Shaopeng Sun, Jiabin Pan, Ning Zhang, Xianghong Jin, Tie-nan Zhu, Zhijie Kang, Jihong Hao, Xiang-dong Li

**Affiliations:** 1 State Key Laboratory of Animal Biodiversity Conservation and Integrated Pest Management, Institute of Zoology, Chinese Academy of Sciences, Beijing 100101, China; 2 University of Chinese Academy of Sciences, Beijing 100049, China; 3 Department of Rare Diseases, Peking Union Medical College Hospital, Chinese Academy of Medical Sciences & Peking Union Medical College, Beijing, China; 4 Department of Hematology, Peking Union Medical College Hospital, Chinese Academy of Medical Sciences & Peking Union Medical College, Beijing 100730, China; 5 Department of Hematology, the Second Hospital of Dalian Medical University; Liaoning Medical Center for Hematopoietic Stem Cell Transplantation; Liaoning Key Laboratory of Hematopoietic Stem Cell Transplantation and Translational Medicine, Dalian, China; 6 Department of Clinical Laboratory, the Second Hospital of Hebei Medical University, Shijiazhuang 050000, China

**Keywords:** MYH9-related disease, nonmuscle myosin IIA, RBC membrane skeleton, red blood cell

## Abstract

*MYH9*-related disease (*MYH9*-RD) is a rare autosomal dominant disorder caused by mutations in *MYH9* gene, which encodes the heavy chain of nonmuscle myosin IIA. Nearly all *MYH9*-RD patients present with macrothrombocytopenia, characterized by decreased platelet count and increased platelet size. In the present study, we collected blood samples from three *MYH9*-RD patients (R702S, D1424N, and R1464C) and unexpectedly found that the actin levels in the red blood cells (RBCs) from all three *MYH9*-RD patients are substantially lower than the healthy controls. We further revealed that the levels of two RBC membrane skeleton proteins, α-spectrin and tropomodulin, are also reduced in *MYH9*-RD RBCs. We showed that the membrane skeleton of *MYH9*-RD RBCs was more porous and that *MYH9*-RD RBCs produced more severe deformation under hyperosmotic pressure compared with healthy controls. We propose that defects in the membrane-skeleton network of RBCs may be an abnormal manifestation of MYH9-RD.

## Introduction

*MYH9*-related disease (*MYH9*-RD) is a rare autosomal dominant disorder caused by mutations in *MYH9* gene [1–3]. Although different *MYH9*-RD mutations have different effects on disease severity and progression, almost all *MYH9*-RD patients develop macrothrombocytopenia, which is characterized by a decrease in platelet count and an increase in platelet size [4]. Additionally, some patients may later develop non-syndromic deafness, nephritis, and cataracts [1,5,6]. A recent study reported that *MYH9* mutations can also lead to abnormalities in red blood cell (RBC) morphology [7].

The *MYH9* gene encodes the heavy chain (HC) of nonmuscle myosin IIA (NMIIA), an actin-based hexameric motor protein composed of two HCs, two essential light chains (ELCs), and two regulatory light chains (RLCs) [8]. Each HC contains a globular motor head that possesses actin-binding and ATPase activities, a lever arm stabilized by one ELC and one RLC, and a C-terminal α-helical tail that dimerizes the two HCs, forming the coiled-coil tail of the hexamer [9,10]. NMIIA is widely expressed and plays a crucial role in a broad range of fundamental cellular processes, including cell migration, cytokinesis, morphological changes, and adhesion [8,11,12]. Over 200 pathogenic mutations associated with NMIIA have been reported, the majority of which are missense mutations. These mutations can be broadly categorized into two main types based on their location within the structural domains: mutations in the motor head and those in the tail region. The most frequent and pathogenic mutations in the motor domain affect Arg702 (R702), which are associated with the most severe and complex symptoms in *MYH9*-RD patients [13–16]. Among the mutations in the tail region, mutations at Asp1424 (D1424) are one of the most prevalent and extensively studied tail mutations [14,15]. While different *MYH9*-RD mutations have been reported to differentially affect the structure and function of NMIIA, it appears that many *MYH9*-RD mutations result in elevated NMIIA activity [12,17–21]. The myosin II inhibitor blebbistatin was reported to rescue the proplatelet formation defects in *in vitro*-generated megakaryocytes from 11 patients with different *MYH9*-RD mutations [22].

Similar to the situation in platelets, NMIIA is the only NMII isoform present in RBCs [7,23]. However, unlike the obvious platelet defects in *MYH9*-RD patients, the symptoms in *MYH9*-RD RBCs are very mild. *MYH9*-RD does not cause clinically significant anemia, and patient RBCs have normal osmotic deformability except for a slight decrease of hemoglobin content and a slight increase in the number of elongated RBCs [7]. RBCs are biconcave discs, and the maintenance of their shape and deformability relies on the membrane skeleton located beneath the plasma membrane [24–26]. The membrane skeleton in RBCs is a highly cross-linked two-dimensional network composed of flexible (α1β1)_2_-spectrin tetramers, which interconnect at junctional complexes formed by short actin filaments (∼37 nm in length) and several associated proteins, including tropomodulin, P4.1, and dematin [24,27–31]. RBC membrane skeleton is regularly organized with a constant proportion of its constituent proteins [29,32]. Smith et al. demonstrated that NMIIA forms bipolar filaments that associate with the membrane cytoskeleton through their motor domains, generating tension via motor activities to control the biconcave disc shape and deformability of RBCs [23]. It was proposed that *MYH9*-RD mutations enhance the association of NMIIA with spectrin-actin membrane skeleton, causing abnormal RBC morphology [7]. However, given that the density of NMIIA filaments in the RBC is much lower than the density of spectrin-actin membrane skeleton, it is puzzling that NMIIA contractility has such a large effect on the shape and membrane properties of RBCs [33].

In the present study, we collected blood samples from three *MYH9*-RD patients (R702S, D1424N, and R1464C) and unexpectedly found that the actin levels in the RBCs from all three *MYH9*-RD patients are substantially lower than normal subjects. We further revealed that the levels of RBC membrane skeleton proteins α-spectrin and tropomodulin are also reduced in *MYH9*-RD RBCs. We showed that the membrane skeleton of *MYH9*-RD RBCs is more porous and that *MYH9*-RD RBCs produce more severe deformation under hyperosmotic pressure compared with normal subjects.

## Methods

### Blood collection

Whole blood was collected from *MYH9*-RD patients or healthy human donors into EDTA tubes at the Second Hospital of Hebei Medical University (Shijiazhuang, China), Peking Union Medical College Hospital (Beijing, China), and the Second Hospital of Dalian Medical University (Dalian, China). Complete blood counts were determined with automated hematology analyzers at the point of collection, and peripheral blood smears were prepared and stained with Wright–Giemsa. Additional EDTA tubes containing whole blood were collected at the same time and delivered within 24 h at 4°C to Institute of Zoology, Chinese Academy of Sciences (Beijing, China), where osmotic fragility assays, RBC isolation, Wright–Giemsa-stained peripheral blood smears, and freeze-etching (see below) were performed within 24 h of arrival. All remaining blood samples were aliquoted into small volumes, flash-frozen in liquid nitrogen, and stored at −80°C. Blood samples were retrieved from patients upon written informed consent, in accordance with the Declaration of Helsinki (approval number of Research Ethics Committee of the Second Hospital of Hebei Medical University: 2022-R030).

Healthy control samples were recruited and collected concurrently with patient samples at each clinical site to minimize batch-to-batch technical variation. The R702S patient, as the first enrolled subject, was matched with a pool of four concurrent healthy volunteers to establish a robust reference baseline. For the D1424N and R1464C patients, each patient was individually matched to one healthy volunteer recruited and sampled at the same time, with age matched as closely as practically possible. All control samples were processed in parallel with corresponding patient samples for all downstream experiments.

### SDS–PAGE and western blot analysis of whole blood and RBCs

The whole blood samples of *MYH9*-RD patients and healthy controls for SDS–PAGE and western blot analysis were prepared as follows. Whole blood was diluted 10 times by mixing 100 μl of whole blood with 900 μl of ice-cold lysis buffer (50 mM Tris pH 7.4, 150 mM NaCl, 1% Triton X-100, 5 mM EDTA, 10 mM NaF, 0.5% sodium deoxycholate, 0.1% SDS, 0.2 mM PMSF, 1× protease and phosphatase inhibitors). After vigorously vortexing, the diluted whole blood sample was incubated on ice for 20 min and then sonicated at 4°C to ensure complete lysis. The lysed sample was mixed with equal volume of 2× SDS loading buffer (100 mM Tris–HCl pH 6.8, 4% SDS, 0.08% bromophenol blue, 24% glycerol, 2% β-mercaptoethanol) and then incubated at 100°C for 5 min. The whole blood samples were aliquoted into small volumes and stored at −80°C.

For SDS–PAGE analysis, 10 μl of whole blood samples or RBC samples were electrophoresed on gradient SDS–polyacrylamide gels (4%–20%), followed by Coomassie brilliant blue staining. For western blot analysis, 10 μl of blood samples were electrophoresed on SDS–polyacrylamide gels (10% gels for actin, tropomodulin, dematin, and P4.1; 6% gels for α-spectrin and NMIIA HC) and then transferred to PVDF membranes for immunoblotting. Primary antibodies were diluted 1000 times, including mouse anti-β-actin (Proteintech, 66009-1), rabbit anti-tropomodulin (Sangon Biotech, D126514), rabbit anti-dematin (Sangon Biotech, D163701), rabbit anti-P4.1 (Solarbio life science, K109011P), rabbit anti-α-spectrin (Solarbio life science, K009298P), and rabbit anti-NMIIA HC (Solarbio life science, K001583P). Secondary antibodies were diluted 10 000 times, including donkey anti-rabbit-HRP (Abcam ab205722) and donkey anti-mouse-HRP (Abcam ab6820). The protein band intensities were measured using ImageJ.

RBCs from *MYH9*-RD patients with D1424N or R1464C mutations were isolated immediately upon arrival. Two milliliters of fresh whole blood was diluted with 2 ml of PBS and then carefully layered over 3 ml of Ficoll-Paque PLUS (Cytiva) in a centrifuge tube. The mixture was centrifuged at 400 × ***g*** for 30 min at 20°C to settle the RBCs at the bottom layer. The RBCs were collected from the centrifuge tube and washed three times in ice-cold PBS by suspending in PBS followed by centrifugation at 600 × ***g*** for 5 min to collect the RBCs. After the final wash, the RBCs were suspended in PBS, and the volume was adjusted to 2 ml, matching the initial volume (2 ml) of the whole blood samples. The isolated RBCs were aliquoted into 100 μl, quick frozen in liquid nitrogen, and stored at −80°C. The RBCs from healthy human donors were processed at the same time as a control. The RBC samples for SDS–PAGE and Western blot were prepared following the same procedures for whole blood, except for using the isolated RBC samples corresponding to 100 μl of whole blood.

### Quick-freeze, deep-etch, and cryo-SEM of RBC membrane skeletons

The whole blood samples were fixed in silicon substrates immediately upon arrival as follows. Whole blood (2 μl) was diluted 100 times by gently mixing with 198 μl of PBS. The diluted whole blood (15 μl) was applied to poly-L-lysine-treated silicon substrates and incubated at room temperature for 30 min to facilitate RBC adhesion. The samples were incubated with 50 mM KPO_4_, pH7.4, 2 mM MgCl_2_, 10 mM EGTA, 1 μM phallacidin, 0.5% Triton X-100, and 0.05% glutaraldehyde at room temperature for 2 min to permeabilize RBCs, and then washed rapidly with wash buffer (50 mM KPO_4_, pH7.4, 2 mM MgCl_2_, 10 mM EGTA, 1 μM phallacidin). Subsequently, the samples were treated with 1% glutaraldehyde in wash buffer for 10 min at 37°C to fix RBC membrane skeletons. After being washed with distilled water, the silicon substrates were quick-frozen and stored in liquid nitrogen.

Deep-etch and replication were performed using Leica EM ACE600 Sputter Coater. The silicon substrates stored in liquid nitrogen were transferred to Leica EM ACE600 Sputter Coater using a cryopreservation transfer system (Leica EM VCT500 Vacuum Cryo Transfer System). The samples were frozen-dried at −84°C for 20 min and metal-shadowed with ∼4 nm of platinum. Finally, the samples were transferred to a cryo-SEM (Zeiss crossbeam 340) for imaging under liquid nitrogen conditions.

Pore size analysis of the RBC membrane skeleton was performed using ImageJ (National Institutes of Health, U.S.A.) on quick-freeze deep-etch electron microscopy images. Measurements were restricted to the central three-quarter region of each RBC to minimize edge deformation artifacts. A fixed threshold value of 90 was uniformly applied across all groups to segment membrane skeleton protein filaments from pore voids, and individual pore area was quantified using the Analyze Particles tool. Only closed, non-overlapping pore structures with well-defined boundaries were included in the analysis; particles falling outside the area range of 0–2.5 × 105 nm^2^ were excluded to eliminate imaging artifacts and false-positive selections. All pore measurement data were exported for subsequent statistical analysis, with statistical results for each group derived from 5 individual RBCs. Identical ImageJ parameters were applied to all images from both control and patient groups to prevent quantification bias.

### Peripheral blood smears

The effects of different osmotic conditions on the morphology of RBCs were analyzed immediately after the arrival of blood samples. The blood samples were treated with different osmotic conditions at room temperature for 30 min and then stained with Wright–Giemsa. The blood smears were imaged using a Nikon ECLIPSE 80i with a 100× oil immersion objective.

Morphological changes were scored using a manually defined four-grade semiquantitative scale, established based on the classic osmotic deformation trajectory of RBCs (transition from biconcave discs to echinocytic morphology, and ultimately to shrunken and fragmented state) combined with our experimental observations. The grading criteria were as follows: (i) Normal RBCs: typical biconcave disc shape with smooth margins; (ii) Mildly deformed RBCs: slightly irregular contours with sparse spicules, with overall disc architecture largely intact; (iii) Moderately deformed RBCs: loss of biconcave structure, presenting as echinocytes with multiple spicules and evident volume shrinkage; (iv) Severely deformed RBCs: fragmented and shrunken morphology with severely contracted membrane structure and complete loss of normal RBC shape. All assessments were performed on 3 independently prepared smears per sample, with 9 random fields (3 fields per smear) analyzed in total; all RBCs in each field were classified into one of the four grades, and the proportion of each grade was calculated for intergroup comparison.

### Statistical analyses

The significance of differences among means was assessed using one-way ANOVA followed by a Bonferroni post hoc test, performed with GraphPad Prism 9 software.

## Results

### Actin levels are reduced in *MYH9*-RD RBCs

To investigate the symptoms caused by *MYH9*-RD mutations, we collected whole blood samples from three *MYH9*-RD patient donors (R702S, D1424N, and R1464C) and healthy human donors simultaneously (demographic details in Supplementary Table S1). The R702S patient was compared with a pool of four concurrent healthy controls, while D1424N and R1464C patients were each compared with one individually matched healthy control recruited at the same time. All three *MYH9*-RD patients exhibited significant enlargement of platelets in Wright–Giemsa stained smears (Supplementary Figure S1) and had reduced platelet counts (Table 1). We analyzed the levels of NMIIA HC and cytoskeleton proteins in the whole blood of the patients using Western blot. Unexpectedly, we found that the actin levels in the whole blood of *MYH9*-RD patients were noticeably lower than those in the healthy controls. When equal amounts of total proteins were loaded for the whole blood samples of the patients and the healthy controls, the actin levels in the whole blood samples of R702S, D1424N, and R1464C patients were approximately 50%, 69%, and 71%, respectively, of those in the controls (Figure 1A,B).

**Figure 1:**
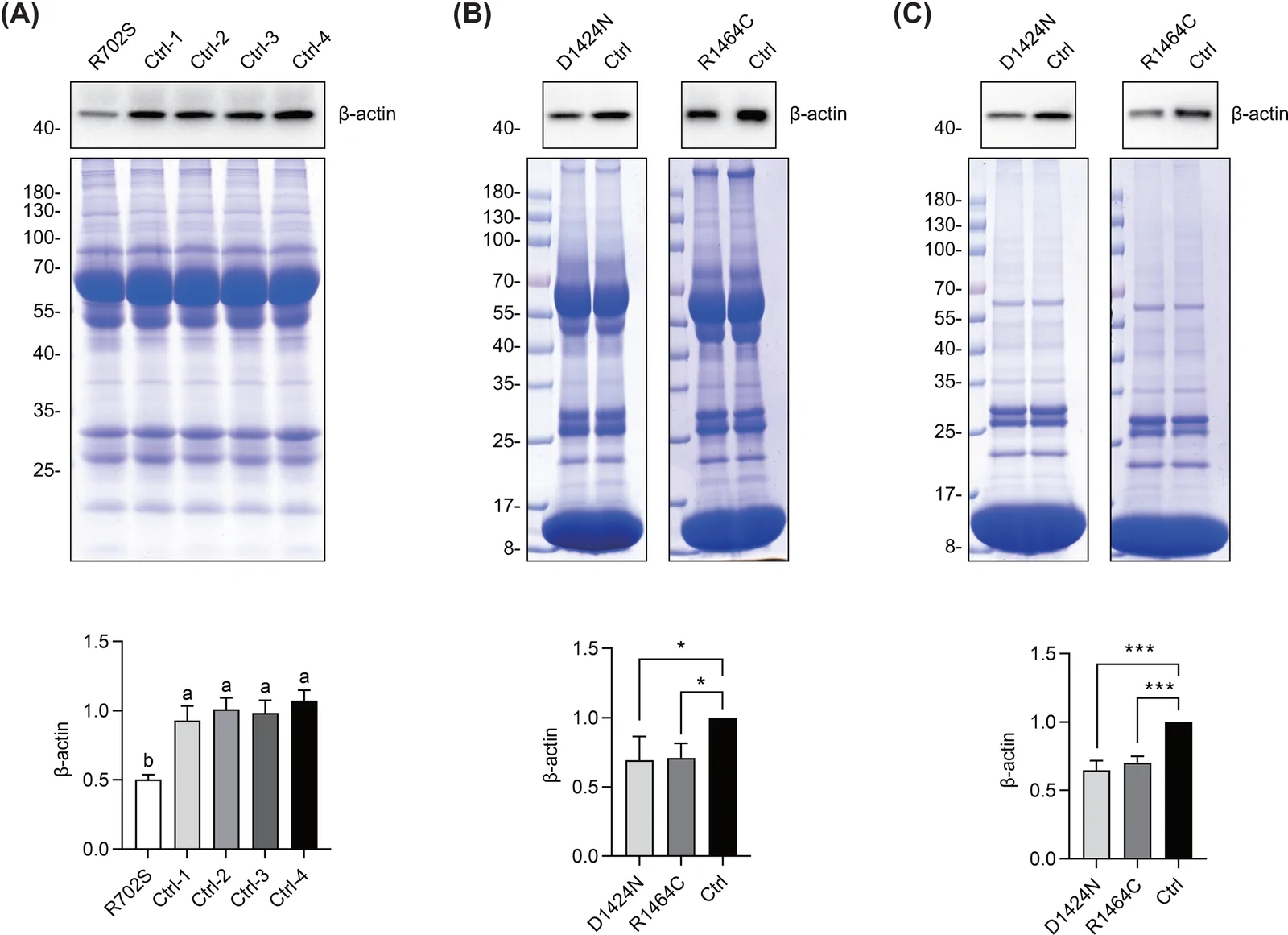
Actin levels in whole blood and RBCs of *MYH9*-RD patients (**A**,**B**) Comparisons of actin levels in whole blood of *MYH9*-RD patients and healthy controls. Actin levels in whole blood were detected by western blot using anti-actin antibody (top), and total proteins were detected by SDS–PAGE and Commassie blue staining (middle). Bottom shows quantification of actin levels in whole blood of *MYH9*-RD patients and healthy controls detected by western blot. The results are the mean ± SD of three independent repeats. (A) The relative levels of actin of R702S patient and healthy controls were normalized to the average value from 4 healthy controls. The statistical differences among the samples were determined using one-way ANOVA with Bonferroni post hoc test (*P* < 0.05). (B) The relative actin levels of D1424N and R1464C patients were normalized to that of the corresponding healthy control. Data are the mean ± SD of three independent experiments with one-way ANOVA with Bonferroni post hoc test (*P < 0.05). (**C**) Comparisons of actin levels in RBCs of *MYH9*-RD patients and healthy controls. Actin levels in RBCs were detected by western blot using anti-actin antibody (top), and total proteins were detected by SDS–PAGE and Coomassie blue staining (middle). Bottom shows quantification of actin levels in RBCs of *MYH9*-RD patients and healthy controls detected by western blot. The relative actin levels of D1424N and R1464C patients were normalized to that of the corresponding healthy control. Data are the mean ± SD of three independent experiments with one-way ANOVA with Bonferroni post hoc test (***P < 0.001).

**Table 1:** CBCs of *MYH9*-RD patients and the reference ranges for healthy individuals

	R702S (♀)	D1424N (♂)	R1464C (♀)	Reference ranges
PLT (×10^9^/l)	24	14	105	125–350
RBC (×10^12^l)	4.84	5.13	4.41	4.0–5.5 (♂), 3.8–5.1 (♀)
WBC (×10^9^/l)	4.63	3.7	9.41	3.5–9.5
MCV (fl)	82.8	92	88.4	82–100
MCH (pg)	27.1	30.5	30.4	27–32
MCHC (g/l)	328	332	344	320–360
RDW (%)	15.8	13.6	12.3	11–16
HCT (%)	40.1	47.2	39	35–50
HGB (g/l)	132	157	134	120–160 (♂), 110–150 (♀)

Note: PLT, platelet count; RBC, red blood cell count; WBC, white blood cell count; MCV, mean corpuscular volume; MCH, mean corpuscular hemoglobin; MCHC, mean corpuscular hemoglobin concentration; RDW, red cell distribution width; HCT, hematocrit; HBG, hemoglobin. ♂, male; ♀, female.

Given that RBCs are the most abundant cell type in blood, we speculated that the actin in whole blood is primarily derived from RBCs and the decrease in actin level observed in the whole blood of *MYH9*-RD patients is mainly due to RBCs. Indeed, Western blotting showed that ∼75% of actin in whole blood is derived from RBCs (Supplementary Figure S2). We then isolated RBCs from the whole blood of D1424N and R1464C patients and analyzed the actin levels using western blot. As expected, we found that the actin levels in both D1424N and R1464C RBCs were significantly lower than in the healthy controls (Figure 1C). Densitometry of the western blot showed that the levels of actin in D1424N and R1464C RBCs were approximately 65% and 70% of that in the healthy human RBCs, respectively (Figure 1C).

Due to lack of isolated R702S RBC samples, we were unable to directly determine the actin level in R702S RBCs. However, we were able to estimate the actin levels in R702S RBCs based on the fact that RBCs contribute ∼75% actin in the whole blood of healthy control and the actin level in the R702S whole blood was reduced to 50% of the normal level. We expected that the actin levels in R702S RBCs were at most 67% of normal levels, even if all actin in the R702S whole blood is derived from the RBCs. Therefore, we conclude that the actin levels are reduced in the RBCs of all three *MYH9*-RD patients, i.e. R702S, D1424N, and R1464C.

We also determined the NMIIA levels in the whole blood and the RBCs of *MYH9*-RD patients by western blot. Compared with the healthy control, the NMIIA levels in the whole blood of *MYH9*-RD patients were significantly reduced, i.e. 58% for D1424N, 33% for R1464C, and 52% for R702S of those in healthy controls (Supplementary Figure S3A). The NMIIA levels in the RBCs of D1424N, and R1464C were 60% and 36%, respectively, of those in healthy controls (Supplementary Figure S3B). We were not able to estimate the NMIIA levels in R702S RBCs, because NMIIA in RBCs constitutes only a minor fraction of the total NMIIA in whole blood (Supplementary Figure S2).

### The levels of several membrane skeleton proteins are reduced in *MYH9*-RD RBCs

In RBCs, actin exists in short filaments with a highly uniform length of ∼37 nm. Short actin filaments and several associated proteins assemble into junctional complexes interconnected by long (α1β1)_2_-spectrin tetramers, forming a two-dimensional cytoskeletal network beneath the plasma membrane of RBCs. Because the stoichiometric ratio of membrane skeletal proteins in RBCs is largely constant, we expected that a reduction of actin levels in RBCs would lead to a decrease in the levels of other membrane skeleton proteins.

To test this hypothesis, we examined the levels of several membrane skeleton proteins, including α-spectrin, tropomodulin, P4.1, and dematin, in the whole blood and RBCs of *MYH9*-RD patients using western blot (Figure 2). In whole blood (Figure 2A,B), α-spectrin levels in the three patients were approximately 75% of those in healthy controls, and tropomodulin levels in R702S, D1424N, and R1464C were approximately 54%, 76%, and 78%, respectively, of those in healthy controls. P4.1 levels in R702S and D1424N were similar to those in healthy controls, whereas that in R1464C was approximately 74% of those in healthy controls. The levels of dematin in three patients were similar to those of healthy controls. In RBCs (Figure 2C,D), α-spectrin levels and tropomodulin levels in both D1424N and R1464C were reduced (76% in D1424N and 73% in R1464C for α-spectrin; ∼74% in both D1424N and R1464C for tropomodulin). The levels of P4.1 and dematin in both D1424N and R1464C were similar to those in health controls. Because α-spectrin and tropomodulin in whole blood are majorly derived from RBCs (Supplementary Figure S2), we inferred that the reduction of those proteins in whole blood of R702S patients originates from RBCs.

**Figure 2:**
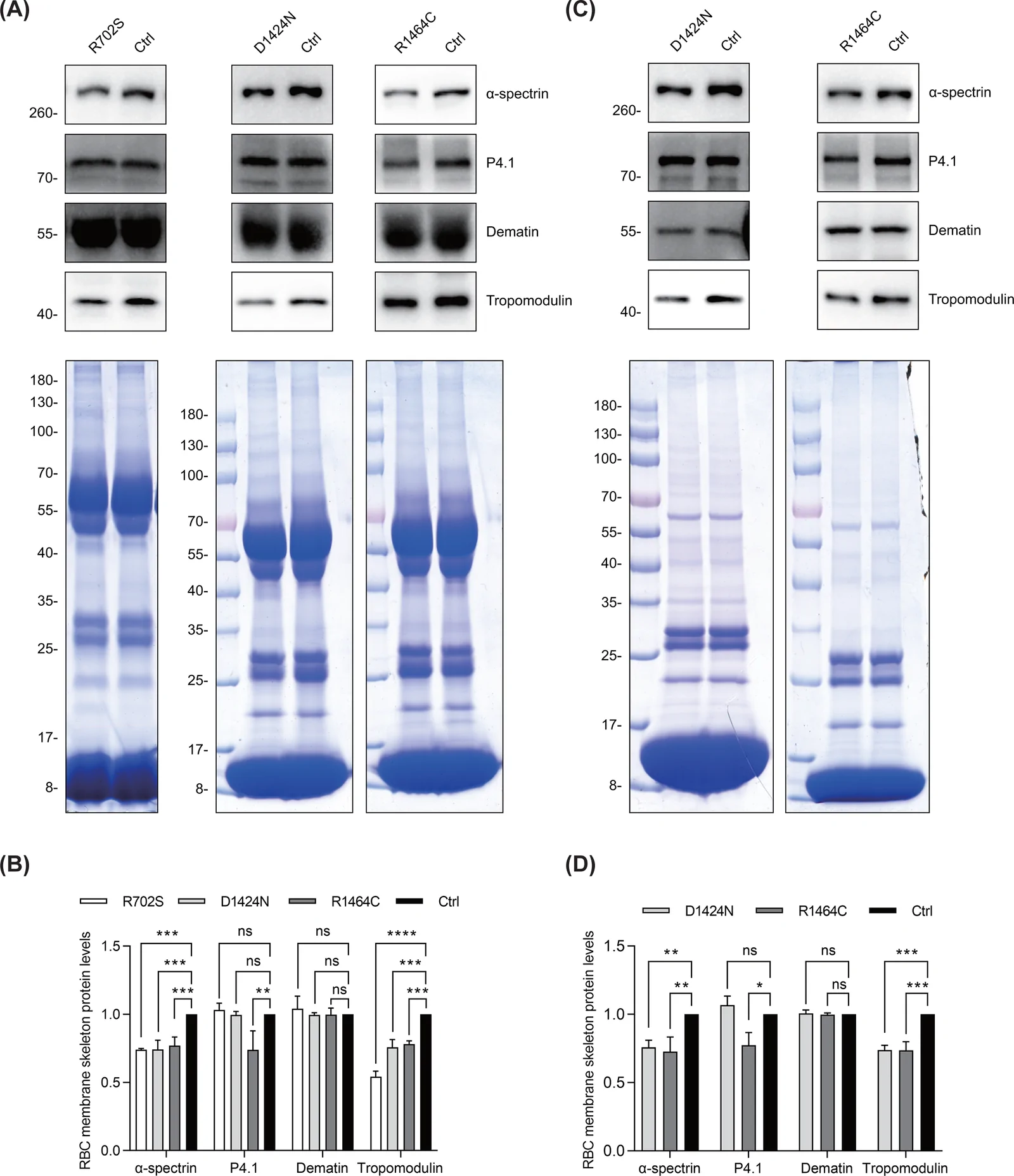
The membrane skeleton protein levels in whole blood and RBCs of *MYH9*-RD patients (**A,B**) Comparison of the levels of RBC membrane skeleton proteins in whole blood of *MYH9*-RD patients (R702S, D1424N, and R1464C) and healthy controls. (A) RBC membrane skeleton proteins (α-spectrin, P4.1, dematin, and tropomodulin) in the whole blood samples were detected by western blot (top), and total proteins were detected by SDS–PAGE and Commassie blue staining (bottom). (B) Relative amounts of RBC membrane skeleton proteins in the whole bloods. The levels of membrane skeleton proteins for patients are normalized to healthy controls. Data are the mean ± SD of three independent experiments with one-way ANOVA with Bonferroni post hoc test (ns, no significance, **P < 0.01, ***P < 0.001, ****P < 0.0001). (**C**,**D**) Comparison of the levels of membrane skeleton proteins in RBCs of *MYH9*-RD patients (D1424N and R1464C) and healthy controls. (C) RBC membrane skeleton proteins (α-spectrin, P4.1, dematin, and tropomodulin) in the RBC samples were detected by western blot (top), and total proteins were detected by SDS–PAGE and Commassie blue staining (bottom). (D) Relative amounts of RBC membrane skeleton proteins in RBCs. The levels of membrane skeleton proteins for patients are normalized to healthy controls. Data are the mean ± SD of three independent experiments with one-way ANOVA with Bonferroni post hoc test (ns, no significance, *P < 0.05, **P < 0.01, ***P < 0.001).

### The membrane skeleton density in *MYH9*-RD RBCs is decreased

Given the reduced levels of several membrane skeleton proteins in *MYH9*-RD RBCs, we expected a lower density or abnormality of membrane skeleton networks in *MYH9*-RD RBCs. To test this possibility, we used quick-frozen, deep-etch, and cryo-SEM technique to examine the membrane skeleton networks in the RBCs of *MYH9*-RD and the healthy controls (Figure 3A). The normal RBCs exhibit a relatively uniform network composed of filaments intersecting at multiple branching points, whereas the membrane skeleton lattice apertures in D1424N RBCs and R1464C RBCs were much larger and more variable than in normal subjects. Quantification of the lattice pore sizes revealed that the average lattice size in normal RBC membrane skeletons was approximately 2342 nm^2^, whereas the lattice pore sizes in D1424N and R1464C RBC membrane skeletons averaged approximately 4481 nm^2^ and 4984 nm^2^, respectively (Figure 3B and Supplementary Figure S4).

**Figure 3:**
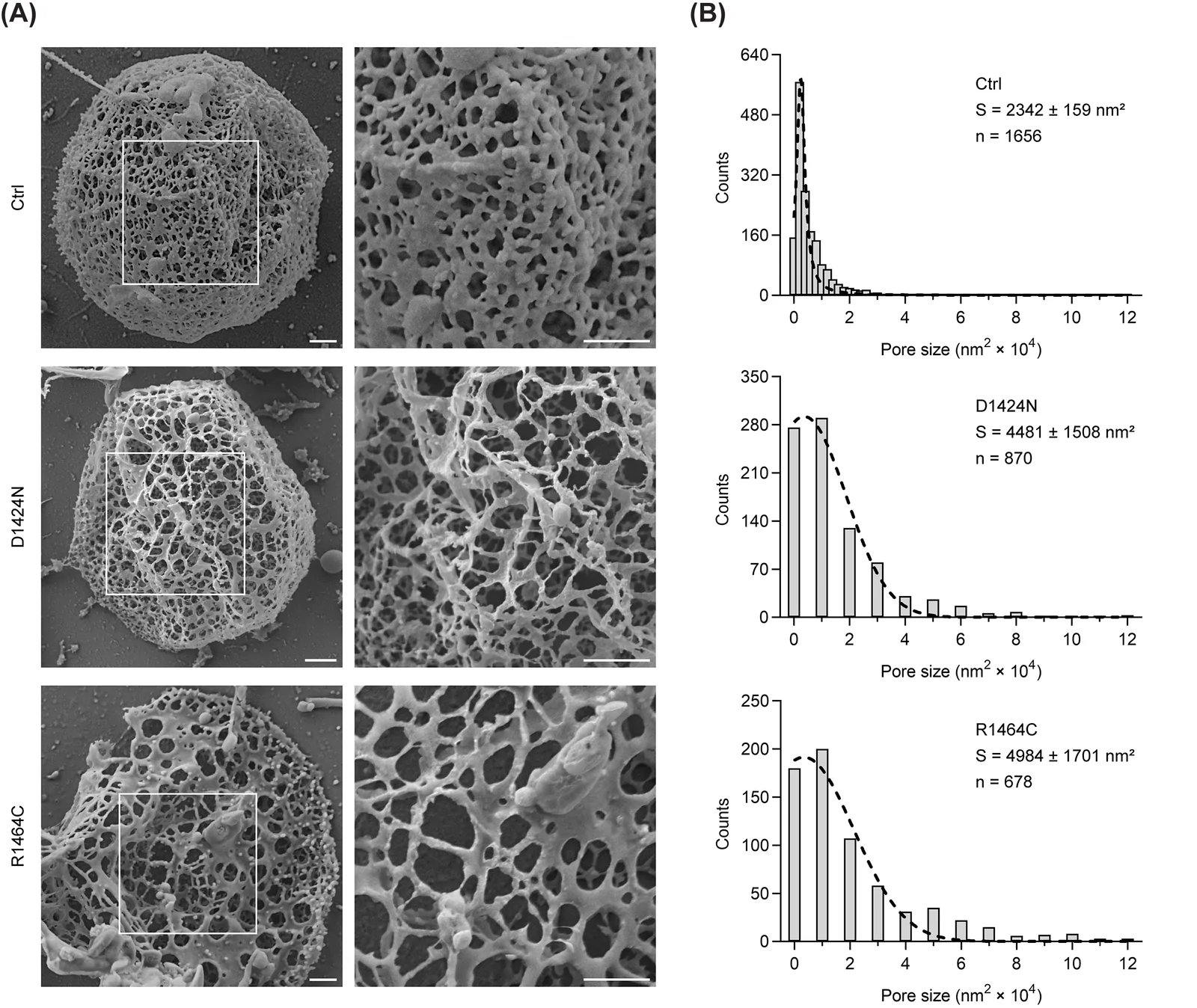
SEM images of RBC membrane skeleton of healthy controls and *MYH9*-RD patients (**A**) SEM images of the quick-frozen, deep-etched RBC membrane skeleton of healthy controls, D1424N, and R1464C. Note that two independent matched healthy control samples were collected and processed in parallel with the two patient samples; both were included in the pore size quantification, and only one representative control micrograph is shown here for conciseness. Scale bars = 500 nm. (**B**) Quantification of lattice pore sizes in the RBC membrane skeletons of healthy controls, D1424N, and R1464C. Dotted lines indicate the fitted frequency distribution curves of lattice pore size for each group. Only the pores located in the central 3/4 region of RBCs were quantified. Statistical data for each group are derived from 5 RBCs. “n” is the number of quantified pores.

### *MYH9*-RD RBCs produce greater deformation under hypertonic conditions compared with healthy controls

The biconcave disk morphology and deformation of RBCs rely on the membrane skeleton. Given the vast impact of the *MYH9*-RD mutations on the components and structure of RBC membrane skeleton, we expected some defects in the morphology and deformability of *MYH9*-RD RBCs. Wright-Giemsa-stained peripheral blood smears showed that approximately 20% of RBCs from both D1424N and R1464C patients exhibited abnormal shape with jagged edge, whereas very few RBCs from healthy controls did (Figure 4A, column 1). We examined morphology of *MYH9*-RD RBCs and healthy controls under different osmotic conditions ranging from 0.675% to 1.8% NaCl (Figure 4). Under hypotonic conditions with 0.675% NaCl, nearly all RBCs remained intact without undergoing osmotic lysis. Notably, abnormal crenated RBCs were seldom observed in both patients, suggesting the previously aberrant morphology may have been partially restored. Surprisingly, after isotonic treatment with 0.9% NaCl, crenated cells in both patients were also seldom observed, suggesting that the morphologically abnormal RBCs may have reverted to a normal shape. Under hypertonic conditions with 1.2% NaCl, both patients’ RBCs, as well as the healthy control, exhibited crenated morphology. Under hypertonic treatment with 1.5% NaCl, nearly all RBCs from the patients and the healthy control exhibited morphological changes. However, the deformation of the *MYH9*-RD RBCs was significantly more severe, with most showing significant damage, whereas healthy RBCs were relatively less damaged. Under extreme hypertonic stress with 1.8% NaCl, both control and patient RBCs showed further aggravated shrinkage and morphological damage, with MYH9-RD RBCs consistently more severely affected. Above observations suggest that the mechanical properties of *MYH9*-RD RBC membrane are compromised, especially resistance to hypertonic pressure is reduced.

**Figure 4:**
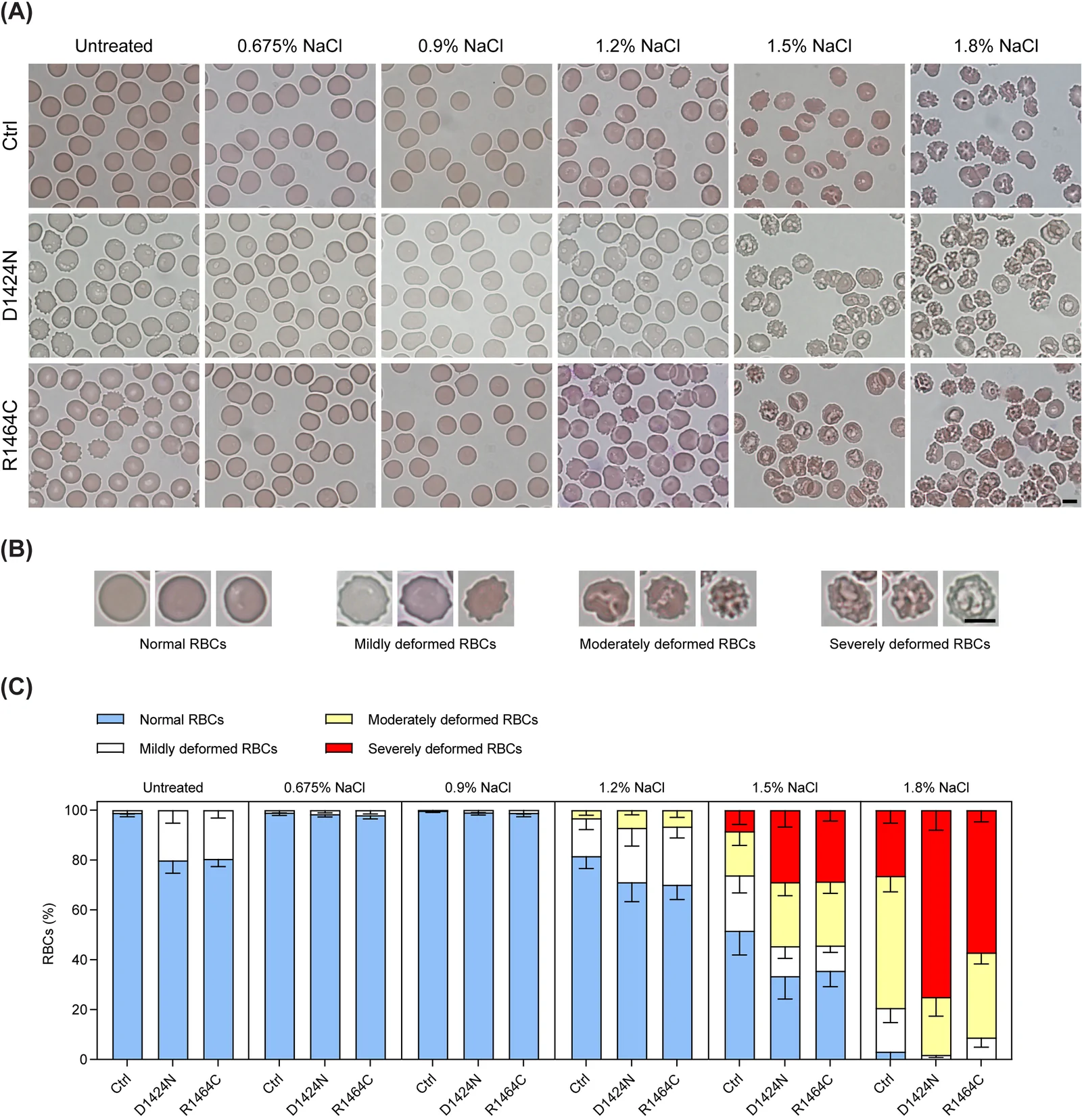
Wright–Giemsa-stained peripheral blood smears from *MYH9*-RD patients and normal subjects under different osmotic conditions (**A**) Representative images of smears from a normal control and *MYH9*-RD patients with D1424N or R1464C mutations under different NaCl concentration conditions. Scale bar = 5 μm. (**B**) Classification of RBC morphology based on the severity of deformation. (**C**) Quantification of RBC morphology under different NaCl concentration conditions.

## Discussion

Typical symptoms of *MYH9*-RD are macrothrombocytopenia and Döhle bodies in leukocytes [1,6]. Here, we discovered a new laboratory feature of *MYH9*-RD: decreased density in the membrane skeleton in *MYH9*-RD RBCs. We found that in all three *MYH9*-RD patients tested (R702S, D1424N, and R1464C), levels of at least three membrane skeleton proteins (actin, α-spectrin, and tropomodulin) in RBCs are substantially decreased. We also found that *MYH9*-RD RBCs have enlarged lattice aperture of membrane skeleton network and abnormal deformability compared with healthy controls.

In the present study, we were able to detect altered actin content in *MYH9*-RD RBCs because our western blot results were normalized to the amount of total proteins rather than to actin level. β-actin is one of the most commonly used loading controls during a western blot, as it is expressed at high levels within most eukaryotic cell types and is generally unaffected by experimental conditions. However, this is not the case for *MYH9*-RD RBCs. As actin levels decreased in *MYH9*-RD RBCs, so did α-spectrin and tropomodulin, consistent with the fact that these two proteins interact closely with actin in the RBC membrane skeleton network. On the other hand, we did not observe a clear reduction of other membrane skeleton proteins such as P4.1 and dematin in *MYH9*-RD RBCs. We expect that those extra membrane skeleton proteins may bind abnormally to actin or diffuse in the cytosol.

A recent study also reported that *MYH9*-RD mutations cause the abnormal morphology of RBCs [7]. However, in contrast with our findings, they found that *MYH9*-RD mutations did not affect the composition and organization of RBC membrane skeleton proteins, including actin and α1-spectrin. This discrepancy may be attributed to the different sample handling and quantification control methods used in the current study as compared with the previous one. Firstly, using SDS–PAGE combined with protein staining (Coomassie blue staining), their study found that RBC ghosts from *MYH9*-RD patients contained normal levels of major membrane skeleton components such as α1/β1-spectrin, protein 4.1R, and actin. In contrast, we used RBCs rather than RBC ghosts as samples to quantify the levels of membrane skeleton proteins in the *MYH9*-RD RBCs. The preparation of RBC ghosts is more tedious and may mask the difference in the levels of membrane skeleton proteins in the RBCs. Further studies directly comparing intact RBCs and purified RBC ghosts will help clarify the source of this discrepancy. Secondly, their total internal reflection microscopy (TIRFM) images of actin and α1-spectrin beneath the plasma membrane show no detectable differences between the normal controls and *MYH9*-RD RBCs, whereas our quick-frozen, deep-etch scanning electron microscopy of RBCs reveals the significantly enlarged lattice aperture in *MYH9*-RD RBCs. The distance between the actin nodes of RBC membrane skeleton network was estimated to be 40 nm to 200 nm [24,34], which cannot be resolved by conventional TIRFM.

The membrane skeleton is crucial for maintaining RBC morphology and deformation, and many RBC membrane diseases lead to abnormal deformability and fragility [23,35]. It is noteworthy that the moderate but substantial reduction of membrane skeleton proteins in *MYH9*-RD RBCs only slightly affects the morphology and deformability of RBCs. RBCs are remarkably flexible and deformable, suggesting that the membrane skeleton network of RBCs is very resilient and may tolerate small defects. Interestingly, the *MYH9*-RD RBCs show fairly normal morphology and slight deformation under normal and hypotonic conditions, but are relatively more sensitive to hypertonic conditions. The RBC membrane skeleton consists of a number of basic mesh-like units, with membrane filaments interconnected to form a meshwork located beneath the cell membrane. The overall structure of RBC membrane skeleton resembles a cage, providing excellent tensile strength but relatively poor compressive strength. From this point of view, it is not unexpected that a moderate reduction in the density of membrane skeleton in *MYH9*-RD RBCs only slightly affects tensile strength, but greatly impairs compressive strength. Nevertheless, chronic and subtle changes in the RBC membrane skeleton—which may lead to long-term complications of MYH9-RD (such as progressive nephropathy) through mechanisms such as alterations in microcirculation or increased RBC fragility—warrant further study.

How *MYH9*-RD mutations lead to the reduction of membrane skeleton proteins in RBCs is a question that deserves further investigation. RBCs develop from committed stem cells through a process called erythropoiesis, which involves the expulsion of the nucleus and other intracellular organelles, as well as most of cytoskeletal structures, and the assembly of the membrane skeleton. It is possible that NMIIA plays a role in the assembly of the RBC membrane skeleton and that abnormal activities of NMIIA mutants in *MYH9*-RD RBCs cause abnormal assembly of membrane skeleton network. *MYH9*-RD mutations affect platelets, leukocytes, and RBCs. All these blood cell types differentiate from common myeloid progenitor cells or more primitive hematopoietic stem cells [36,37]. It has been reported that *MYH9*-RD mutations can destabilize the folded state of NMIIA, making it more prone to adopting an active state [21]. This suggests that *MYH9*-RD mutations may lead to increased activities of NMIIA, potentially resulting in the premature differentiation of RBCs before proper maturation. This could also explain how abnormal proplatelet formation contributes to the macrothrombocytopenia and the rescue effects of blebbistatin [18,19,22]. Taken together, we hypothesize that the proper assembly of membrane skeleton in RBCs depends on the normal activity of NMIIA and abnormal activities of NMIIA mutants in *MYH9*-RD RBCs cause the abnormal assembly of membrane skeleton network.

Based on the current findings from the study of three *MYH*-RD mutations, we propose that the defects in RBC membrane skeleton revealed in the present study may represent a new laboratory feature of *MYH9*-RD. However, given the potential influence of age on RBC membrane properties, our findings should be interpreted with caution. To date, more than 100 *MYH9*-RD mutations have been identified. Further validation in larger, well-matched patient cohorts will be necessary to determine the general prevalence of these abnormalities across different mutation subtypes.

## Supplementary Material

Supplementary Figures S1-S4 and Table S1

## Data Availability

For original data, please contact lixd@ioz.ac.cn.

## Abbreviations

ELCs: essential light chains
HC: heavy chain
MYH9-RD: MYH9-related disease
NMIIA: nonmuscle myosin IIA
RBCs: red blood cells
RLCs: regulatory light chains
TIRFM: total internal reflection microscopy
