# Supplementary material for: The membrane skeleton density of red blood cells in MYH9-related disease patients is decreased
Source: Biosci Rep. 2026 Sep 29;46(10):BSR20260386. doi: 10.1042/BSR20260386 (PMC13623819; doi:10.1042/BSR20260386)
Supplement: Supplementary file 1 [file BSR-2026-0386_supp.pdf]

Supplemental Data

Figure S1

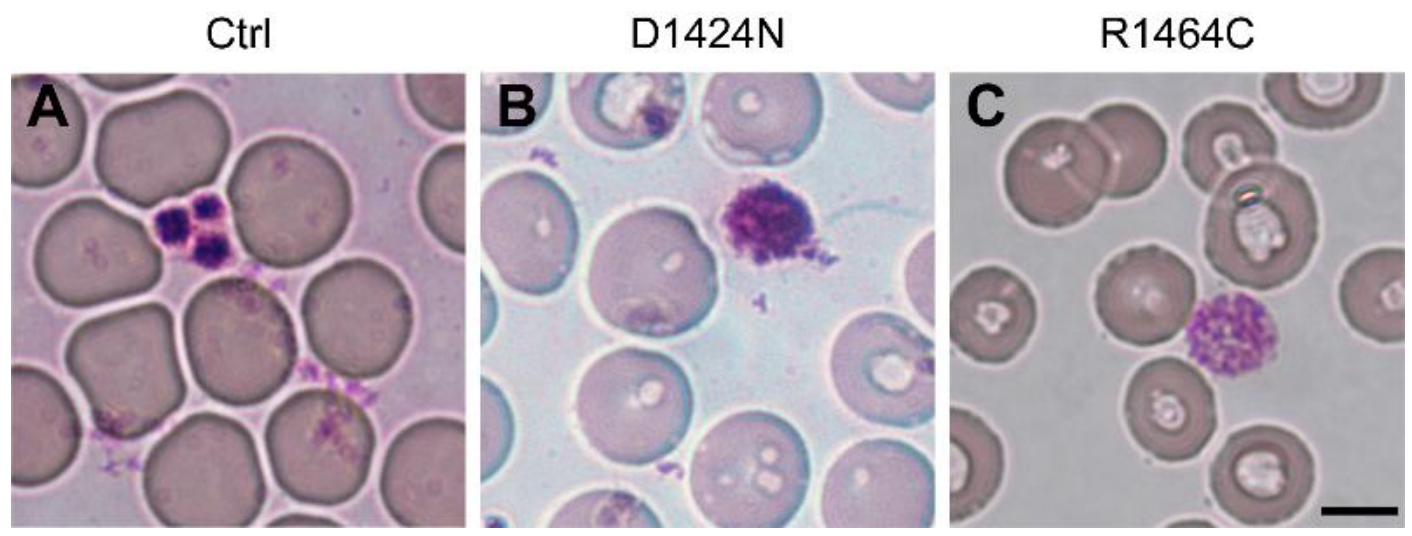

Figure S1. Wright-Giemsa-stained peripheral blood smears from healthy control, D1424N, and R1464C. The *MYH9*-RD patients exhibit significant enlargement of platelets. Scale bar = 5 μm.

Figure S2

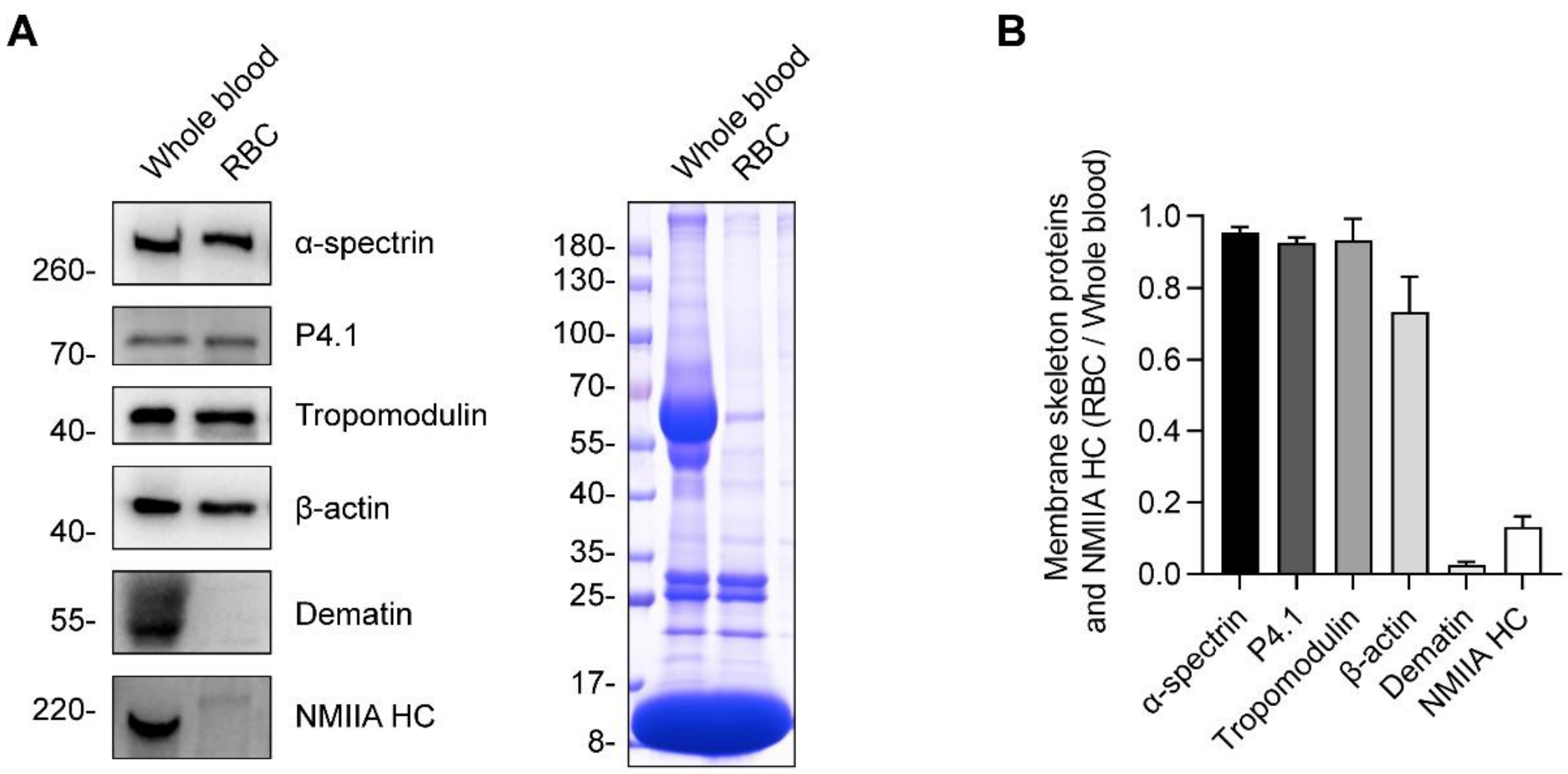

Figure S2. Proportions of membrane skeleton proteins and NMIIA HC in RBCs relative to their levels in whole blood. Analyses of the proportions of membrane skeleton proteins and NMIIA HC in RBCs relative to their total levels in whole blood, showing that with the exception of dematin and NMIIA HC, which are expressed at very low levels in RBCs, the remaining membrane skeleton proteins account for the vast majority of these proteins in whole blood. (A) Western blot (left panel) of membrane skeleton proteins and NMIIA HC in whole blood and in RBCs. Note that RBCs are isolated from whole blood, with the final volume adjusted to match that of the original blood sample. SDS-PAGE (right panel) of total proteins of whole blood and RBC samples used in left panel is served as loading control. Equal volumes are loaded for CBB staining. (B) Quantification of Western blot in (A). The ratios were calculated by dividing the band intensity in RBCs by the corresponding band intensity in whole blood. Data are the mean ± SD of three independent experiments.

Figure S3

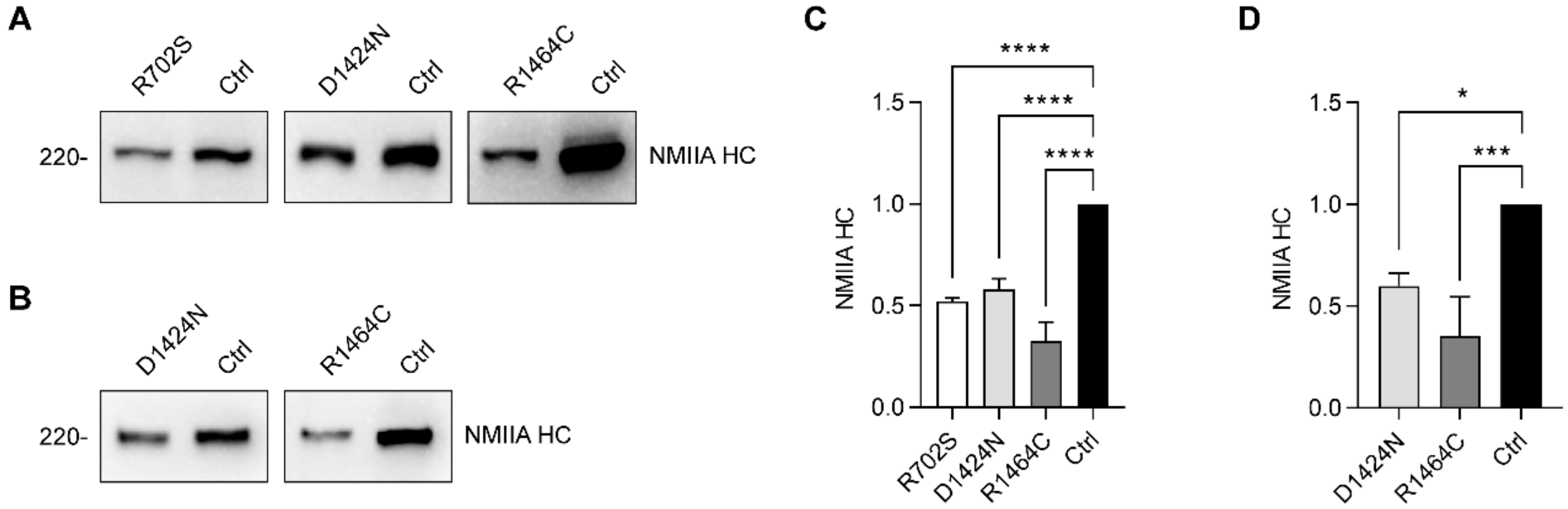

Figure S3. The levels of NMIIA HC in *MYH9*-RD patients are reduced. (A) Western blot of NMIIA HC in whole blood samples from *MYH9*-RD patients and healthy controls, showing NMIIA level is reduced in *MYH9*-RD whole blood compared to controls. (B) Western blot of NMIIA HC in RBCs from D1424N, R1464C and healthy controls, demonstrating NMIIA levels are reduced in D1424N and R1464C RBCs compared to healthy controls. The loaded proteins in SDS-PAGE with Coomassie blue staining are shown in Figure 2A and 2C. (C) and (D) Quantification of western blot in (A) and (B), respectively. The levels of NMIIA HC for patients are normalized to the values from healthy controls. Data are the mean ± SD of 3 independent experiments with one-way ANOVA with Bonferroni post hoc test (*$P < 0.05$, ***$P < 0.001$, ****$P < 0.0001$).

Figure S4

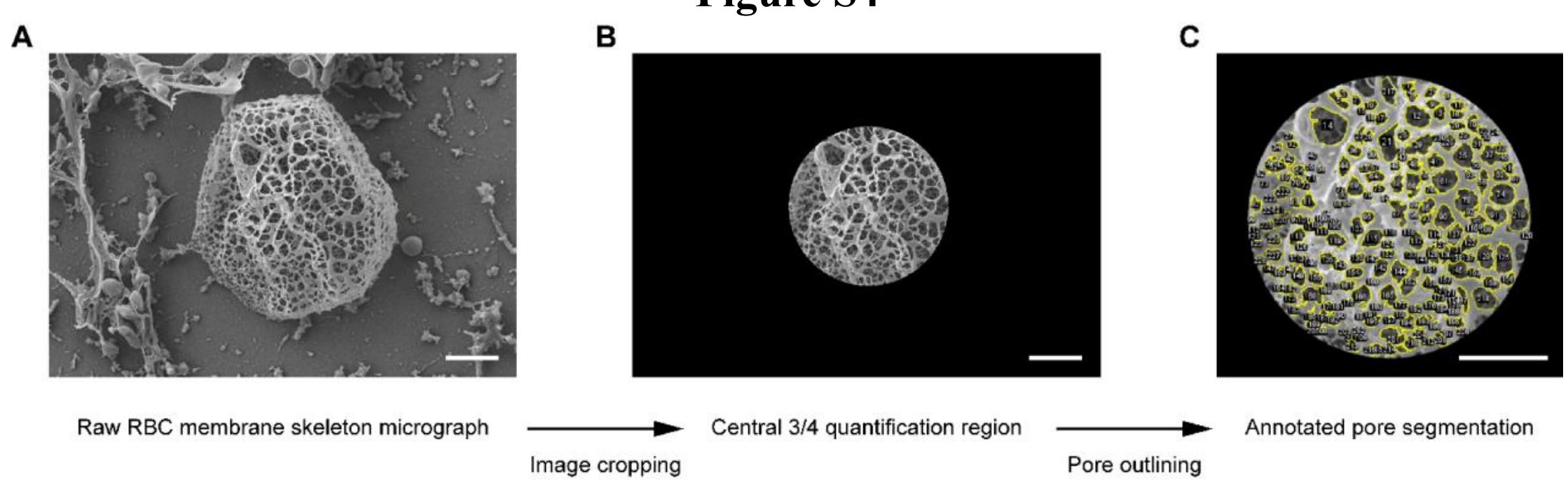

Figure S4. Representative example of RBC membrane skeleton pore size analysis workflow. (A) Original quick-freeze deep-etch electron micrograph of the RBC membrane skeleton. (B) Cropped central three-quarter region of the same cell, used for pore size quantification to avoid edge artifacts. (C) The same region with annotated pore boundaries (yellow outlines), demonstrating the criteria for closed, well-defined pore structures included in the analysis. Numbers inside the pores represent pore IDs used for tracking and recording individual pore area data. This example uses a representative D1424N sample also shown in Figure 3 to demonstrate the analysis pipeline. Scale bars = 1 μm.

Table S1. Demographic information of enrolled subjects

Cohort	Subject ID	Sex	Age (yr)
R702S cohort	R702S	Female	17
	Ctrl-1	Female	24
	Ctrl-2	Male	27
	Ctrl-3	Female	24
	Ctrl-4	Male	23
D1424N cohort	D1424N	Male	22
	Ctrl (matched)	Male	28
R1464C cohort	R1464C	Female	52
	Ctrl (matched)	Female	42

Note: Healthy control samples were collected concurrently with patient samples. All ages refer to the age at the time of blood sample collection.
